# Spatial Analysis, Influencing Factors, and Source-Oriented Probabilistic Health Risks of Potential Toxic Elements in High Geological Background Soil in Central and Southern Shandong Peninsula, China

**DOI:** 10.3390/toxics13110945

**Published:** 2025-11-03

**Authors:** Fang Wan, Xiuwen Zhang, Yan Li, Shenglin Liu, Jianwei Li, Chuang Zhao, Lin Zhang, Yanhong Lou, Zeqiang Sun

**Affiliations:** 1 State Key Laboratory of Nutrient Use and Management, Institute of Agricultural Resources and Environment, Shandong Academy of Agricultural Sciences, Jinan 250100, China; wanfang202010@163.com (F.W.);; 2 Shandong Institute of Geophysical and Geochemical Exploration, Jinan 250100, China; 3 Shandong Agricultural Technology Promotion Center, Jinan 250100, China; 4 Colleges of Resources and Environment, Shandong Agricultural University, Tai’an 271018, China

**Keywords:** soil potential toxic elements, high geological background, source-oriented, health risk assessment, Monte Carlo simulation, positive matrix factorization, Shandong

## Abstract

This study investigates the accumulation, influencing factors, sources, and health risks of eight potential toxic elements (PTEs) in soils from the central–southern Shandong Peninsula, a region characterized by a high geological background and intensive human activities. Concentrations of Cr, Cd, Cu, Ni, Pb, Zn, As, and Hg were analyzed in 19,484 topsoil samples. The results showed that Cr, Cu, and Ni levels exceeded national background values, primarily linked to basalt distribution. Utilizing positive matrix factorization (PMF), spatial analysis, and comparative assessment, four primary sources were identified: natural sources (36.79%), combined traffic and agricultural activities (34.20%), coal combustion (17.32%), and industrial emissions (11.69%). A health risk assessment indicated that while non-carcinogenic risk was within the acceptable limits for the general population, it exceeded the threshold for children in 2.53% of cases, with As from coal combustion being the predominant contributor. These findings provide a critical theoretical basis for implementing targeted, source-oriented control strategies to mitigate PTE pollution in areas where high geological background and anthropogenic activities intersect.

## 1. Introduction

Soil contamination by potentially toxic elements (PTEs) poses a significant global environmental threat. Their ubiquity, combined with their persistent, toxic, and bioaccumulative nature, jeopardizes ecosystem stability [1,2,3,4]. The extensive environmental accumulation of PTEs, including cadmium (Cd), chromium (Cr), copper (Cu), nickel (Ni), lead (Pb), zinc (Zn), arsenic (As) and mercury (Hg), causes soil quality degradation and food chain contamination, thereby posing significant threats to human health [5,6,7]. While anthropogenic activities, including industrial emissions, mining operations, agricultural practices, and automobile exhaust, are extensively documented as primary contributors to PTEs pollution, regions characterized by elevated geological backgrounds exhibit distinct environmental challenges. These challenges are marked by high concentrations of PTEs, an extensive regional distribution, and pronounced spatial heterogeneity. Therefore, it is of great significance to study the enrichment pattern and accumulation characteristics of PTEs in soil derived from the high-geological-background areas.

Recent studies have demonstrated that geogenic contributions can surpass anthropogenic inputs in areas characterized by naturally elevated metal concentrations [8,9,10]. The concentrations of PTEs in soil exhibit strong geochemical correlations with the composition of parent rock. During pedogenesis, ongoing weathering processes concentrate metallic elements from the bedrock, ultimately leading to regions with elevated geogenic background levels [11,12,13]. Globally, four predominant lithologies are characteristic of regions with high geological background levels: (1) black shales, (2) ultrabasic rocks, (3) basic rocks, and (4) carbonate rocks. Black shales, notably prevalent in Europe, North America, China, and the Korean Peninsula, are distinguished by their high organic matter content and are typically enriched with PTEs such as As, Cd, and Cr, attributable to their unique depositional environments and diagenetic histories [14,15,16]. Ultrabasic rocks, primarily composed of serpentine and peridotite, are significantly enriched in Ni, Cr, and cobalt (Co). These lithologies constitute approximately 1% of the Earth’s surface area and are predominantly located along tectonic plate boundaries [17,18,19]. As the most common basic rock, basalt significantly contributes to elevated concentrations of Cr and Ni in derived soils. These metal-enriched basaltic soils are widely distributed across major continental flood basalt provinces, particularly in the Deccan Traps (India), the Columbia River Plateau (United States) and the Emeishan Basalt Formation (China) [20,21,22]. Carbonate rocks constitute approximately 12% of Earth’s continental surface area and their weathering products are typically characterized by significant Cd enrichment in the resulting soils [23,24]. Previous research has established that the spatial distribution patterns of PTE in soils are fundamentally governed by the parent rock [25]. The mechanisms underlying the enrichment of PTE in soils due to the weathering of parent rock are complex. It has been demonstrated that climatic conditions and topography significantly influence weathering rates, thereby impacting the accumulation of PTEs in soils and sediments [26]. Recent studies indicate that Fe-Mn oxides play a critical role in Cd enrichment during carbonate rock weathering [27], whereas Fe oxides are primarily responsible for Cr enrichment during basalt weathering [21,28]. Thus, the type and distribution of parent rocks are primary factors governing the spatial heterogeneity of PTEs in soil. Beyond these natural factors, land use types also exert a significant influence, representing a substantial anthropogenic impact. For example, recent studies in the northeastern Qinghai–Tibet Plateau have shown that construction land and farmlands present moderate to high ecological risks due to PTEs contamination, with industrial emissions identified as the primary anthropogenic source [29]. In summary, the migration of PTEs from parent rock to soil in areas with a high geological background is affected by the type of parent rock, degree of weathering, geographical conditions, and human activities. However, the complex interplay of geochemical processes governing the enrichment of PTE remains inadequately understood.

Conventional health risk assessments of soil PTEs, following paradigms such as those from the US Environmental Protection Agency, have traditionally used total concentrations as the primary metric. While the integration of the bioavailable fraction represents a significant refinement, we posit that these assessments may still be inadequate because they do not differentiate the toxicity of metals derived from different sources. Elements present in low concentrations but posing high risks in source-specific risk assessments warrant particular attention [30]. For instance, a study on soil PTEs in Guangdong Province revealed that traffic emissions contributed the most to PTE concentrations, yet were not the predominant source of health risks [31]. A growing amount of contemporary research has integrated source apportionment analyses into health risk assessments, transitioning from total concentration-based evaluations to source-oriented health risk assessments [32,33,34]. Moreover, due to variations in age, weight, gender, and metabolic parameters among individuals, the use of fixed-value parameters can lead to inaccuracies in risk assessment. To address this issue, Monte Carlo simulation has been primarily employed in probabilistic health risk assessments to quantify uncertainty [31,35,36]. The integration of source analysis, probabilistic simulation, and health risk assessment enhances the reliability and accuracy of risk evaluations.

The China National Environmental Monitoring Centre (CNEMC) established the first set of national soil element background values in 1990. These values were defined as the natural concentrations of 61 elements (e.g., Hg, Cd, Pb, Cr, As, Cu, Zn, Ni) in soils minimally impacted by human disturbance. The sampling strategy was designed with soil types as the fundamental unit, while also accounting for factors such as parent rock, topography, land use type, and industrial layout. A stratified grid method was adopted, with sampling densities varying by region: one sample per 900 km^2^ in the east, from two to three samples per 2500 km^2^ in the central region, and one sample per 6400 km^2^ in the west. This campaign resulted in the excavation of 4095 representative soil profiles, each with a depth of 1.2 m. Samples were collected from each genetic horizon, and 61 indicators from the A-horizon were statistically analyzed to determine the national background values [37]. Building upon this national framework, a higher-resolution (1:250,000 scale) land quality geological survey was conducted in Shandong Province. In this survey, a total of 39,794 surface soil samples (0–20 cm depth) were collected by compositing five sub-samples at each grid node, with an overall density of one composite sample per square kilometer. The geochemical background values of 54 elements/parameters were subsequently measured [38], establishing local reference benchmarks for the province and laying the foundation for future environmental risk assessments and land quality management.

In regions with high geological backgrounds, geological complexity and intense anthropogenic pressure lead to multiple sources and compounded risks of potentially toxic elements (PTEs) [39]. Central–southern Shandong is a typical region, where intricate geological conditions and intensive industrial activities coexist. Notably, it also serves as a crucial grain-producing region in China. Thus, it is of great significance to quantitatively assess the respective contributions of human activities and natural factors to potentially toxic elements and their associated human health risks in this area. Thus, it is of great significance to clarify the impact of human activities and natural factors on human health risks in this area. Current research on the enrichment of PTEs in high-geological-background areas is constrained by several limitations: (1) limited sample sizes, (2) insufficient research on influencing factors of spatial heterogeneity, and (3) a lack of cross-validation between methods. Consequently, it is imperative to systematically investigate the source characteristics, driving factors, and human health risks through extensive sampling and multiple methods.

**Figure 1:**
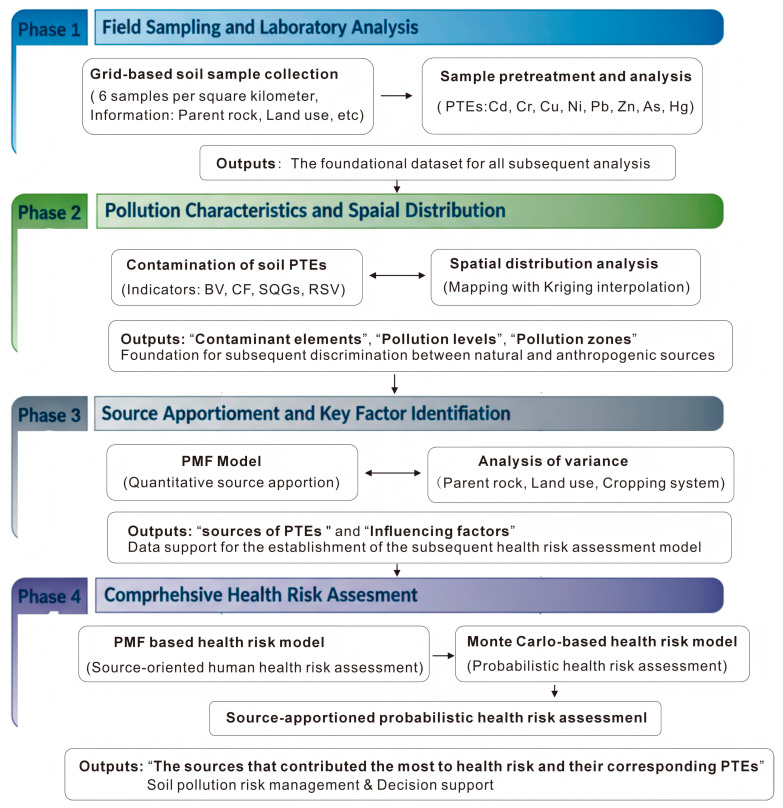
Schematic diagram of the research workflow.

The primary objectives of the present research are as follows: (1) to investigate the concentrations of eight PTEs in soil and compare these values with regional background levels; (2) to analyze the sources of PTEs, utilizing spatial analysis and the positive matrix factorization (PMF) model; (3) to further identify the key influencing factors of specific PTEs by examining parent rock types, land use patterns, and cropping systems; and (4) to conduct a quantitative health risk assessment based on source attribution and probability analysis. A schematic overview of this research framework is provided in Figure 1. The findings of this study aim to provide both theoretical and practical insights for the prevention and management of PTE pollution in regions characterized by high geological background levels.

## 2. Materials and Methods

### 2.1. Research Area

The study area is situated in the hilly regions in central and southern Shandong Province, with geographic coordinates ranging from 118°11′ to 119°06′ east longitude and 35°36′ to 36°13′ north latitude. The topography is predominantly hilly, with an average elevation of 235 m above sea level. The region exhibits a temperate monsoon climate, with an average annual temperature of 14.2 °C and average annual precipitation of 682 mm. The study area is characterized by northeast-trending geological faults with well-developed magmatite formations. Neoarchean granite and diorite are prevalent in the central region of the study area. In the western and northeastern parts, carbonate formations from the Cambrian and Ordovician periods are exposed. Pyroclastic rock formations from the Cretaceous period were found in the eastern and central regions, while Neogene basalt was distributed in the northern parts of the study area. The primary industries in this region include food processing, chemical production, equipment manufacturing, textiles, and mining. This area is also a significant grain-producing region, primarily cultivating wheat and maize.

### 2.2. Sampling Collection and Preparation

**Figure 2:**
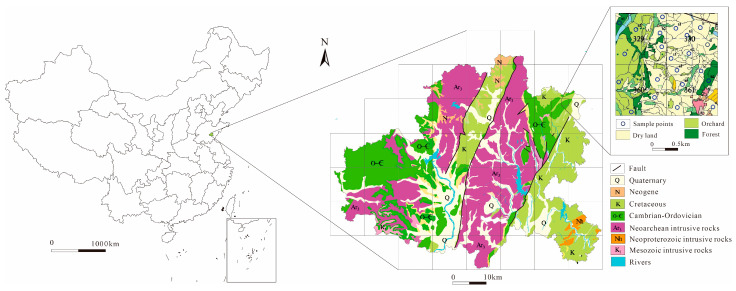
Location of the study area (sample sites in 4 km^2^ as an example).

This study is based on 19,484 soil samples collected during the annual wheat and maize harvest stage between 2018 and 2020 (Figure 2). Sampling was conducted using a grid method, with six samples collected per square kilometer. At each sampling site, 1500 g of surface soil (0–20 cm) was collected in self-sealing polyethylene bags and transported to the laboratory. The soil samples were air-dried at room temperature, and then visible organic residues (e.g., roots, leaves) and coarse particles (>2 mm, including sand and stone fragments) were removed to homogenize the fine soil fraction for subsequent chemical analysis. The air-dried soils were gently ground and passed through a 200-mesh sieve (aperture size: 75 µm) to obtain a homogeneous fine powder for subsequent chemical analysis.

### 2.3. Sample Chemical Analysis

The concentrations of Cu, Zn, Cr, Ni, and Pb in soil samples were quantified using x-ray fluorescence spectrometry (XRF), while the total Cd content was determined via inductively coupled plasma mass spectrometry (ICP-MS). The concentrations of Hg and As were measured using hydride generation atomic fluorescence spectrometry (HG-AFS). Soil pH was assessed using a soil-to-water ratio of 1:2.5, employing a pH meter (PHS-3C, Shanghai, China). The method detection limits (MDLs) were as follows: Cd: 0.03 mg/kg; Cr: 5 mg/kg; Cu: 1 mg/kg; Ni: 2 mg/kg; Pb: 2 mg/kg; Zn: 4 mg/kg; As: 1 mg/kg; and Hg: 0.0005 mg/kg. Quality assurance and quality control (QA/QC) procedures were implemented using blank and duplicate samples, based on standard reference materials (GBW07412, GBW07417) provided by the Center of National Standard Reference Material of China. The results indicated that both the relative standard deviation (RSD) and the logarithmic deviation (ΔlogC) were within 8%, with recovery rates ranging from 92% to 100%.

### 2.4. Contamination Factor (CF)

The CF was calculated according to the following formula [40]:

(1) CF=CsCbackground

where C_s_ is the concentration of PTE in the sample (mg kg^−1^); C_background_ is the concentration of PTE in the background soil (mg kg^−1^). The background values for PTEs in Shandong Province soils are as follows (in mg/kg): Cd, 0.13; Cr, 62; Cu, 22.6; Ni, 27.1; Pb, 23.6; Zn, 63.3; As, 8.6 and Hg, 0.031 [38]. The contamination is classified as CF < 1: low degree; 1 ≤ CF < 3: moderate degree; 3 ≤ CF < 6: considerable degree; and CF ≥ 6: very high degree [41].

### 2.5. Sediment Quality Guidelines (SQGs)

To assess sediment toxicity, the U.S. National Oceanic and Atmospheric Administration (NOAA) established effects-based Sediment Quality Guidelines (SQGs): the Effects Range-Low (ERL) and the Effects Range-Median (ERM) [42]. The ERL and ERM represent the concentrations below and above which adverse biological effects rarely and frequently occur, respectively.

### 2.6. Positive Matrix Factorization Model (PMF)

The USEPA PMF 5.0 model was applied for quantitative source apportionment of PTEs [43]. This receptor model decomposes the original data matrix into factor contribution and factor profile matrices under non-negative constraints [44,45,46], utilizing the uncertainty of each concentration measurement as an input parameter to weight the data points [47,48]. The basic principle is to decompose the original matrix x_ij_ into matrices g_ik_ and f_jk_ and a residual matrix e_ij_. It can be expressed as follows:

(2) $$x_{ij}=\sum_{k=1}^{p}g_{ik}f_{kj}+e_{ij}$$

where x_ij_ represents the concentration of heavy metal j in sample i, p is the number of sources, g_ij_ is the related contribution of factor k to sample i, f_kj_ is the concentration of heavy metal j in source k, and e_ij_ is the residual error matrix. PMF model minimizes the value of the objective function Q in the equation as follows:

(3) $$Q=\sum_{i=1}^{n}\sum_{j=1}^{m}{\left(\frac{e_{ij}}{u_{ij}}\right)}^{2}$$

where u_ij_ is the uncertainty of heavy metal j in the sample i. If the concentration of heavy metal is greater than its corresponding MDL value, the uncertainty is calculated by Equation (4); otherwise, the uncertainty follows Equation (5):

(4) $$Unc=\sqrt{{\left(Errofraction\times concentration\right)}^{2}+{\left(0.5\times MDL\right)}^{2}}$$

(5) $$Unc=\frac{5}{6}\times MDL$$

### 2.7. Source-Oriented Probabilistic Health Risk Model

To quantify the source-specific health risks, the PMF results were incorporated into a source-oriented health risk assessment framework, following established methodologies [49,50,51]. This study integrated the PMF model with a health risk assessment to quantify the contribution of specific PTE sources to potential health risks. This approach was implemented in two steps: first, the source apportionment of PTEs in each soil sample was quantified using the PMF model. Second, the health risks from each contamination source were quantitatively assessed as follows:

(6) $$C_{jn}^{l}=*C_{jn}^{l}\times C_{j}$$

Here, C^l^_jn_ is the mass contribution (mg kg^−1^) of PTE n from source l in the sample j; *C^l^_jn_ is the calculated contributions of PTE n from source l in sample j; and C_j_ is the concentration (mg kg^−1^) of soil PTEs in sample j.

The study evaluated three primary exposure pathways: direct soil ingestion (ing), inhalation of soil particles (inh), and dermal absorption through skin contact (dermal). The average daily exposure doses (ADD^l^_jn,i_) of PTEs through intake, inhalation, and exposure were calculated using Equations (7)–(9), as adapted from the US Environmental Protection Agency [52].

(7) $${ADD}_{jn,ing}^{l}=\frac{C_{jn}^{l}\times {IR}_{ing}\times EF\times ED}{BW\times AT}\times 10^{-6}$$

(8) $${ADD}_{jn,inh}^{l}=\frac{C_{jn}^{l}\times {IR}_{inh}\times EF\times ED}{PEF\times BW\times AT}$$

(9) $${ADD}_{jn,dermal}^{l}=\frac{C_{jn}^{l}\times SA\times AF\times ABS\times EF\times ED}{BW\times AT}\times 10^{-6}$$

where C^l^_jn_ is the concentration of the j th metal in the nth sample from the l th source (mg kg^−1^ day^−1^); IR_ing_ and IR_inh_ is the ingestion rate of soil ingestion (mg day^−1^), the inhalation rate of soil (m^3^ day^−1^) and the ingestion rate of food (mg day^−1^), respectively; SA is the exposed surface area of skin (cm^2^); AF is the adherence factor (kg cm^−2^ day^−1^); ABS is the dermal absorption factor (unitless); PEF is the emission factor (m^3^ kg^−1^); EF is defined as the exposure frequency (day year^−1^); ED is defined as the exposure duration (year); BW is the body weight of the exposed individual (kg); AT is average time of exposure to contaminated soils (day); and 10^−6^ is a unit conversion factor. Details of parameters applied in the exposure assessment model are given in Table S1.

Non-carcinogenic hazards from PTEs are expressed by the hazard quotient (HQ), which is defined by the quotient of the ADD of each PTE and the corresponding reference dose (RfD). The RfD for each PTE is given in Table S2. The hazard indexes (HI) were calculated by the following formula:

(10) $${HI}_{jn}^{l}=\sum {HQ}_{jn,i}^{l}=\sum \frac{{ADD}_{jn,i}^{l}}{{RfD}_{i}}$$

where HQ^l^_jn,i_ is the hazard quotient on the I th exposure pathway from source l of the PTE n in sample j. A probabilistic health risk assessment, integrating the PMF-modeled sources with Monte Carlo simulation, was performed to quantify uncertainties. The corresponding parameters are listed in Tables S3 and S4.

### 2.8. Statistical Analysis

Statistical analyses were performed using WPS Office 2025. The source apportionment of potentially toxic elements (PTEs) was conducted using the US EPA Positive Matrix Factorization (PMF) 5.0 model. A probabilistic health risk assessment incorporating Monte Carlo simulations was performed with Oracle Crystal Ball (version 11.1.3), with 10,000 iterations at a 95% confidence level. Spatial distribution was mapped in ArcMap (version 10.2) using Kriging interpolation. A statistical analysis of parent rock, land use type, and planting system was performed across all sampling locations. Graphs were generated in OriginPro 2025 to illustrate the outcomes of the analysis of variance (ANOVA) with post hoc tests, analyzing the effects of the three primary categorical variables: parent rock, land use type, and cropping system. All figures were finalized for publication using CorelDRAW 2025.

## 3. Results

### 3.1. Concentration and Contamination of Soil PTEs

Table 1 presents the descriptive statistics of PTE concentrations in the soil samples. The mean concentrations (in mg/kg) of Cd, Cr, Cu, Ni, Pb, Zn, As, and Hg were 0.13, 85.0, 26.0, 36.2, 26.8, 68.4, 7.6, and 0.030, respectively. In the study area, the average concentrations of Cr, Cu, Ni, and Pb exceeded both the Chinese soil background values and the local background values for Shandong Province. The contamination factor (CF) calculations indicated moderate contamination (1 ≤ CF < 3) by Cd, Cr, Cu, Ni, Pb, Zn, and Hg, while the remaining PTEs exhibited low contamination levels (CF < 1). According to the sediment quality guidelines (SQGs) presented in Table 1, the mean concentrations of all PTEs in the study area were below the Effects Range-Median (ERM) values. However, the mean concentrations of Cd, Cr, and Ni exceeded their respective Effects Range-Low (ERL) values. These results suggest that most PTEs pose a low risk of adverse biological effects, with the exception of Cd, Cr, and Ni, which may cause occasional to moderate adverse effects. Specifically, 63%, 5.86%, 2.05%,4.72%, 0.54%, 0.29%, 0.48%, and 0.02% of soil samples exceeded the risk screening value of agricultural soils in China (CEPA GB15618-2018) for Cd, Cr, Cu, Ni, Pb, Zn, As, and Hg, respectively. It is worth noting that 18% of soil samples were three times higher than the risk-screening value for Cr and Ni. The above results suggested that the soil in a certain area was contaminated by PTEs in the study region.

### 3.2. Spatial Distribution of PTEs

**Figure 3:**
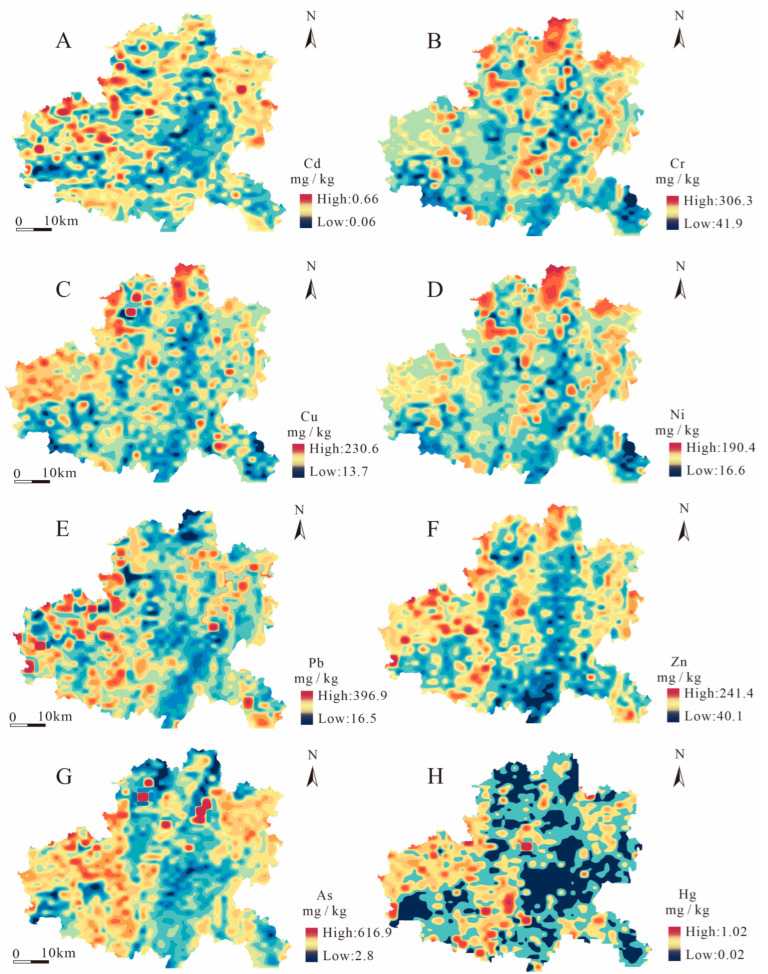
Spatial distribution of concentrations of Cd (**A**), Cr (**B**), Cu (**C**), Ni (**D**), Pb (**E**), Zn (**F**), As (**G**), and Hg (**H**).

The kriging interpolation method was applied for a spatial distribution analysis of each PTEs. The results indicated that the spatial distribution of all PTEs was heterogeneous, while some elements showed strong spatial correlation. Specifically, the elevated Cd contents were distributed in the northwest and northeast sectors (Figure 3A). Cr content was characterized as high in the middle–north and low in the south (Figure 3B). Cu and Ni had a similar distribution pattern to Cr, with enrichment zones clustered in the north (Figure 3C,D). Pb, As and Hg anomalies exhibited a “hot spot” pattern. The maximum content of Pb was observed in the western regions, while the minimum content was found in the central areas (Figure 3E). Zn exhibited that the elevated contents were strip-spread in the eastern, western, and central areas (Figure 3F). The distribution of As demonstrated that the anomalies clustered in the northeast and western zones (Figure 3G). Hg distribution showed that the maximum content was scattered in south-central and northwest (Figure 3H).

The spatial distribution of Ps revealed a coupling relationship with parent rocks. According to the similarity of spatial distribution patterns, PTEs were divided into three categories. The first category included Cr, Cu, and Ni, whose contents were mainly concentrated in the northern part of the study area, which was consistent with the outcrop distribution of basalt in the area, indicating that these elements are closely related to basalt. The second category included Cd, Pb, As, and Hg. The enrichment zones mainly clustered in the northwest of the study area, which was relatively consistent with the exposed range of carbonate rocks in the area, indicating that their distribution is closely related to carbonate rocks. The third type was Zn element. The spatial distribution of elevated contents was strongly associated with carbonate rocks and basalt, suggesting it is attributed primarily to these parent materials. The distribution of PTEs revealed two dominant influences. First, the regional patterns were strongly influenced by parent rocks, leading to the co-enrichment of geochemically affiliated elements. Second, distinct anomalies of Cd, Cu, Pb, As, and Hg were identified spatially adjacent to urban areas, highlighting localized anthropogenic inputs.

### 3.3. Source Apportionment

**Figure 4:**
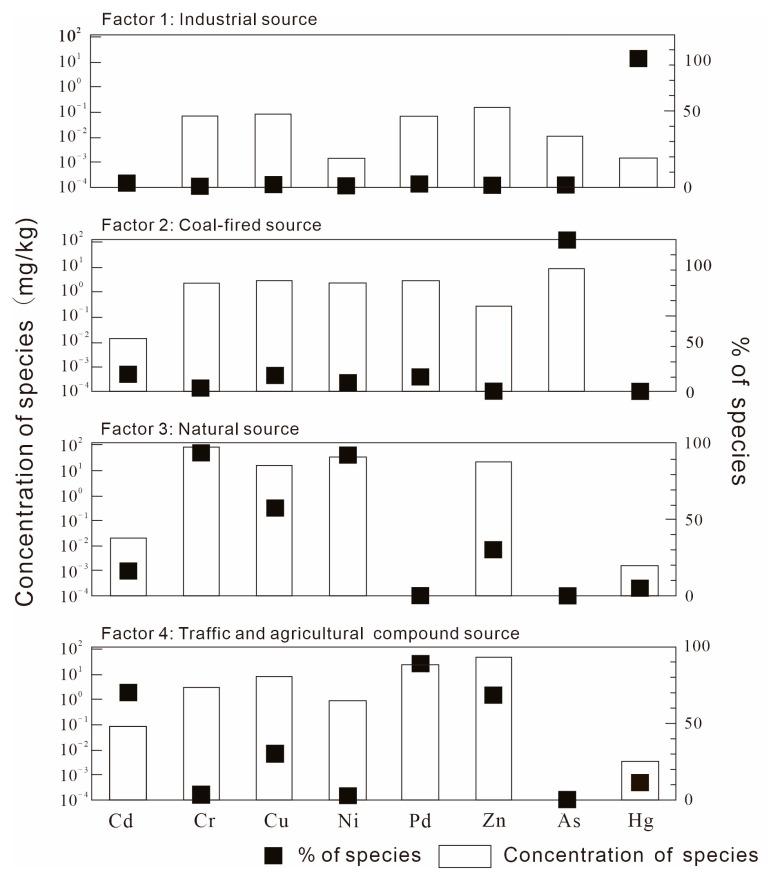
Factor profiles of potential toxic elements by PMF model.

The PMF 5.0 model was applied to identify pollution sources and quantify their contributions. Model inputs included the measured concentrations of PTEs and their associated uncertainties. To determine the optimal solution, models with factor numbers ranging from 2 to 6 were computed, each with 20 bootstrap runs. The selection of the optimal factor number was guided by the following criteria: (1) the objective function Q value and the theoretical value Q have minimal difference; (2) as the number of factors increases, the objective function Q value is at its minimum and stable; (3) the residual value of most soil samples was in the range of −3~3 [43,54,55]. In the current study, the starting seed number was chosen randomly, and the number of runs was set to 20. The most robust solution was achieved with the number of factors set to four, as indicated by a minimum and stable Q value. The resulting concentration profiles and source contributions are presented in Figure 4.

Factor 1 (11.69% of the total contributions) had a strong positive loading on Hg (83.60%). It was found that the average content of Hg in construction land is 0.057 mg/kg, which is significantly higher than that in other land use types in our study. In addition, the spatial distribution of Hg contents indicated that higher values were mainly found in the south–central and northwest part of the study area, which was consistent with the distribution of mining areas and smelters. This region possesses abundant mineral resources. Historical iron and gold mines are situated in the central area (e.g., Zhuge Town and Gaoqiao Town) and the west (e.g., Quanzhuang Town). The smelting facilities are primarily concentrated in the southern vicinity of the city. These industrial activities, especially metal mining and smelting, are significant sources of Hg emissions. The released Hg subsequently accumulates in the surrounding soil via atmospheric dust deposition [56,57,58]. In conclusion, factor 1 was mainly derived from industrial activities.

Factor 2 (17.32% of the total contributions) had strong positive loading on As (98.54%). As is a ubiquitous, highly toxic, and volatile trace element in coal. It can be released into the environment during coal mining, storage, and combustion, causing pollution. In the study area, discrete areas of high arsenic concentration are located in the northern part. Their spatial distribution coincides with the locations of coal storage yards and coal-fired power plants. The investigation found that there was a history of small-scale coal mining in Shagou town in the northern part of the study area. An exceptionally high As concentration of 1880.2 mg/kg was found in the surface soil around the coal preparation plant in Yangzhuang town in the north of the study area. The atmospheric subsidence around coal-fired power plants was the primary source of the enrichment of As in soil [59,60]. Therefore, factor 2 could be regarded as a coal-fired source.

Factor 3 (36.79% of the total contributions) showed strong positive loading for Cr (93.59%), Ni (91.56%), and Cu (57.35%), with a minor contribution from Zn (30.37%). Generally speaking, the contents of Cr and Ni in soil are closely related to the weathering of parent rock. The landform of the study area is mainly a hilly area, with Neogene basalt and Cretaceous pyroclastic rocks exposed in the north. Through the coupled effects of weathering and pedogenic processes, a large-scale geochemical high-background area characterized by elevated Cr and Ni concentrations has formed in this region. According to previous studies, parent rock weathering had a close relation with the high contents of Cr, Ni and Cu [61,62,63]. Therefore, factor 3 was mainly derived from natural sources.

Factor 4 (34.2% of the total contributions) had a strong positive loading on Pb (88.51%) Cd (69.63%) and Zn (68.03%), with a weak positive loading on Cu (30.23%). It was found that the long-term application of phosphate fertilizer and livestock manure led to a significant accumulation of Cd, Cu, and Zn in soil [64,65,66]. In our study, the mean Cu concentration in apple orchards (253 sampling sites) reached 35.4 mg/kg, significantly exceeding the regional average level of 23.8 mg/kg. This phenomenon was largely attributed to the widespread use of copper-based pesticides, which were commonly employed in agricultural practices to control plant diseases and enhance crop yield [67]. Furthermore, Pb contamination was primarily associated with the combustion of vehicular fuels, as evidenced by its strong correlation with traffic density, serving as a characteristic tracer of traffic-derived pollution [68]. The high value of these elements had an overlapping area in the west part of the study area, around the main traffic arteries. Additionally, according to previous studies, Cd and Zn typically accumulated in roadside soils, due to the wearing of tires and brake pads [69]. Therefore, factor 4 was classified as deriving from a mixed source of traffic and agriculture.

In summary, the PMF model identified four sources of PTEs: industrial, coal-fired, natural, and a mixed source associated with transportation and agricultural activities. The natural source factor was the largest contributor among the identified sources. Cu and Zn were apportioned to both natural and anthropogenic sources, with Cu dominated by lithogenic inputs and Zn influenced more by traffic emissions and agricultural practices.

### 3.4. Determination of Key Factors on PTEs Accumulation in Soil

Analysis of variance (ANOVA) followed by Tukey’s HSD test was employed to examine the differences in soil PTE concentrations among different groupings: parent rocks (representing natural sources), land use types (representing anthropogenic sources), and planting patterns (representing agricultural sources).

#### 3.4.1. Parent Rocks

**Figure 5:**
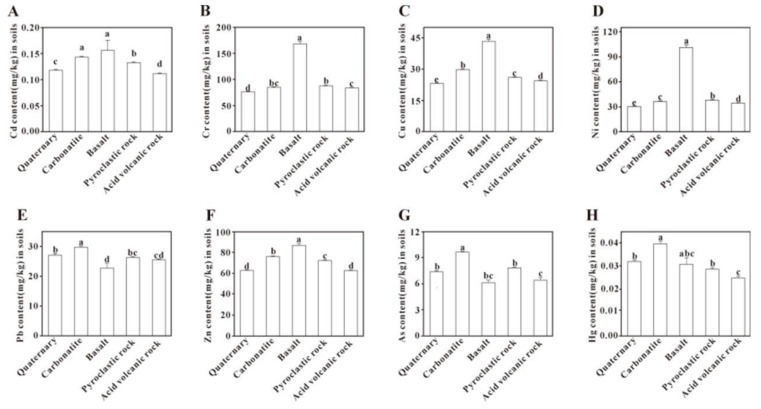
Comparison of potentially toxic element content in soils derived from different parent rocks. Bars labeled with different lowercase letters are significantly different from each other (*p* < 0.05, Tukey’s HSD test).

It was found that Cr, Cu, Ni, and Zn were significantly higher in the soil from basalt than other parent rocks (*p* < 0.05) in Figure 5. Pb and As were slightly higher in the parent rock soil of carbonate rocks than other parent rocks (*p* < 0.05). The weathering of basaltic rocks releases elements such as Cr, Cu, and Ni from primary minerals like olivine and pyroxene, and these elements can be incorporated into secondary clay minerals [21,70]. In contrast, the enrichment of PTEs during carbonate rock weathering follows a different pathway. For example, studies in karst areas of southwest China indicate that Pb enrichment in soils is closely associated with its release upon the oxidation of sulfide inclusions (e.g., arsenopyrite and galena) within weathered calcite and dolomite [71]. Similarly, As is also globally enriched in soils through carbonate weathering processes [72]. Therefore, the composition of parent rock is a key controlling factor in PTEs enrichment, with basalt being the primary source for Cr, Cu, and Ni, and carbonate rocks for Pb and As.

#### 3.4.2. Land Use Type

**Figure 6:**
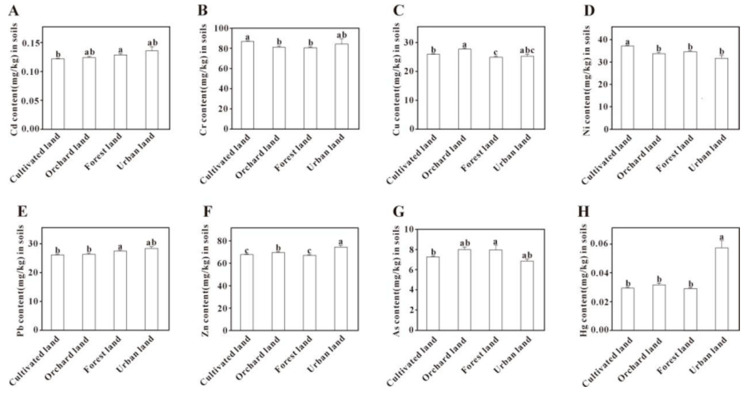
Comparison of potentially toxic element content in soils under different land uses. Bars labeled with different lowercase letters are significantly different from each other (*p* < 0.05, Tukey’s HSD test).

Significant differences in concentrations of PTEs were observed among the different land use types in Figure 6. Specifically, the contents of Cr, Cu, Ni, and Zn were significantly higher in cultivated and orchard soils compared to forest soils (*p* < 0.05). Notably, Cu concentration was the most affected, with its level in orchard soils being substantially greater than that in both cultivated and forest soils. This finding is consistent with previous studies, which reported that land use, particularly in apple orchards and vineyards, has a pronounced impact on the accumulation of Cu and Zn [73]. The soil Hg content in construction land was significantly higher than in other land use types (*p* < 0.05). This elevated concentration is likely a legacy of the traditional chlor-alkali industry, which used mercury as an electrolytic cell catalyst (the mercury process). Although this technology has been phased out in China, historical production may have led to mercury leakage or waste residue accumulation. Subsequently, Hg can percolate into the soil via wastewater and waste residues [74]. Therefore, these findings suggest that industrial activities are the primary source of Hg, which is consistent with Factor 1, identified in the PMF model as the industrial source.

#### 3.4.3. Cropping System

**Figure 7:**
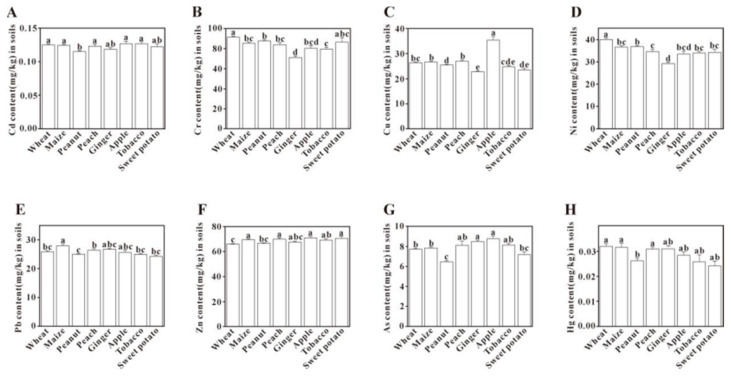
Comparison of potentially toxic element content in soils under different cropping systems. Bars labeled with different lowercase letters are significantly different from each other (*p* < 0.05, Tukey’s HSD test).

The concentrations of individual PTEs varied significantly depending on the crop type in Figure 7. Notably, the Cu content in apple orchards was significantly higher than in all other cropping systems (*p* < 0.05). In contrast, ginger fields exhibited significantly lower levels of Cr and Ni compared to other crop fields. Conversely, no significant differences were detected in the concentrations of Cd, Cu, As, and Hg between wheat and maize fields. Although the parent rock was identified as the primary source of Cu in the PMF analysis (Factor 3), agricultural activities (Factor 4) were also a significant contributor. The notably high Cu content in the apple orchards is therefore likely the result of this mixed origin, with a natural background from parent material being augmented by long-term agricultural practices. This interpretation is strongly supported by global studies which document that elevated Cu in orchards and vineyards stems from the cumulative application of copper-based fungicides over time [75,76].

### 3.5. Concentration-Oriented Health Risk Assessment Base on PMF

The PMF model was applied to quantify the contribution of each source factor to every sample. These quantitative contributions were then used to assess the source-specific health risks of PTEs at each sampling point. The analysis resolved four primary sources: an industrial source, a coal combustion source, a natural source, and a composite traffic–agricultural source. The non-carcinogenic risks of PTEs from different sources to human health were calculated (Table 2 and Table 3). The mean hazard index (HI) values for children (4.96 × 10^−1^) and adults (1.12 × 10^−1^) were both below the safety threshold of 1, indicating no significant non-carcinogenic health risk to the local population. Specifically, the non-carcinogenic risk (HQ) values for children were in the order of As (3.3 × 10^−1^) > Pb (9.9 × 10^−2^) > Cr (3.0 × 10^−2^) > Ni (2.3 × 10^−2^)> Cu (8.4 × 10^−3^) > Zn (2.9 × 10^−3^) > Cd (1.8 × 10^−3^) > Hg (1.3 × 10^−3^). For adults, the order was Cr (5.7 × 10^−2^) > As (3.7 × 10^−2^) > Pb (1.3 × 10^−2^) > Ni (2.8 × 10^−3^) > Cu (9.8 × 10^−4^) > Cd (6.8 × 10^−4^) > Zn (3.6 × 10^−4^) > Hg (2.0 × 10^−4^). These results demonstrate that As and Cr had the greatest effects on the non-carcinogenic risk for children and adults, respectively. The non-carcinogenic risks identified in this study were relatively low compared to those reported in other regions with high geological backgrounds or agricultural products. For instance, in the black shale-rich LouShao Basin, the hazard index (HI) for Cd alone exceeded 1, signaling a notable health risk [77], a level not reached for any element in the present study. Furthermore, the HI values for wheat and rice grains in basalt-derived agricultural soils were as high as 1.60 and 3.09, respectively [20], which are substantially higher than the cumulative risks (HI < 1) observed in our soil samples. These comparisons underscore the lower magnitude of health risks in the current study area.

**Figure 8:**
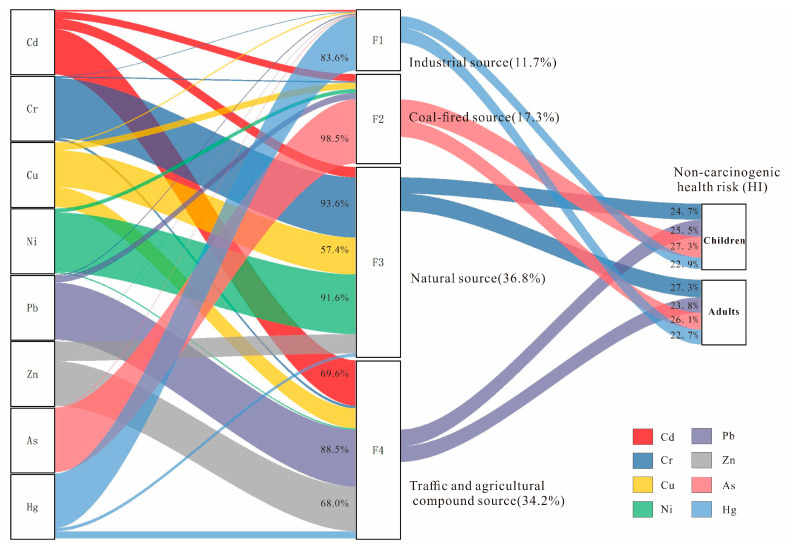
Contributions of different sources to health risks from soil PTEs.

The contribution of each PMF-identified source to the non-carcinogenic health risk was quantified in Figure 8. The ranking of these contributions differed between populations. For children, the order was coal-fired source (27.3%) > traffic and agricultural source (25.5%) > natural source (24.7%) > industrial source (22.9%). In contrast, for adults, the natural source (27.3%) was the dominant contributor, followed by the coal-fired (26.1%), traffic and agricultural (23.8%), and industrial (22.7%) sources. The contributions of the four sources to non-carcinogenic risk were broadly similar. However, the coal-fired source was the dominant contributor for children, while the natural source was the primary contributor for adults. This pattern aligns with the established principle that health risk is more closely associated with the toxicity of PTEs than their total concentration. For instance, natural sources accounted for the largest mass contribution (36.8%) but a lower share of the risk in children (24.7%), which is attributable to the lower toxicity of the associated metals (Cr, Ni, Cu). Conversely, coal combustion sources, representing only 17.3% of the total mass, contributed the highest proportion to children’s health risk (27.3%), due to the high toxicity of arsenic (As). Consequently, pollution control measures should prioritize industries related to coal combustion. This finding is consistent with studies in karst high-geological-background areas in China, where arsenic, despite not being the most abundant contaminant, was identified as the largest contributor to health risks [78].

### 3.6. Probabilistic Health Risk Assessment

**Figure 9:**
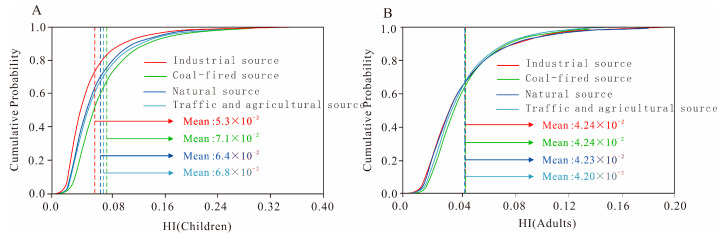
Non-carcinogenic probabilistic health risk assessment for each source ((**A**) for children, (**B**) for adults).

**Figure 10:**
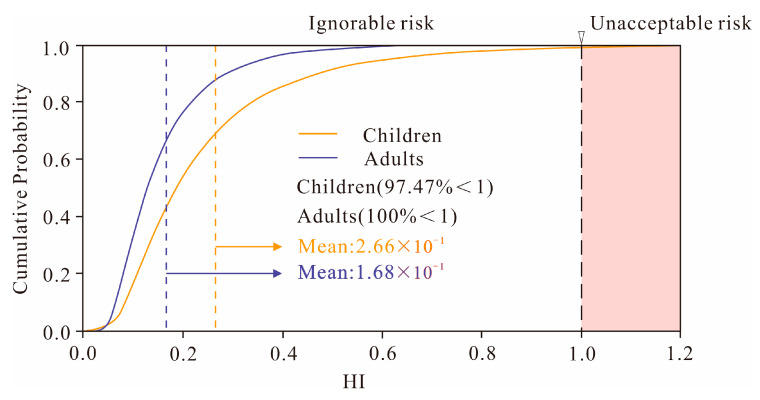
Non-carcinogenic probabilistic health risk assessment.

The non-carcinogenic health risks for children and adults from different PTE sources were evaluated using a Monte Carlo simulation (Figure 9 and Figure 10). The probability distributions revealed that the non-carcinogenic risks from each individual source remained below the safety threshold of 1 for both age groups. Specifically, for children, the mean non-carcinogenic risks decreased in the following order: coal-fired source (7.1 × 10^−2^) > traffic and agricultural source (6.8 × 10^−2^) > natural source (6.4 × 10^−2^) > industrial source (5.3 × 10^−2^), indicating that the coal-fired source was the primary contributor. For adults, the mean risk values across the different sources were all similar, approximately 4.2 × 10^−2^, which is well below the acceptable level. These results demonstrate that PTEs from each source are unlikely to cause non-carcinogenic health effects in the local population.

The probability distribution of the non-carcinogenic risk revealed a mean Hazard Index (HI) of 2.66 × 10^−1^ for children and 1.68 × 10^−1^ for adults. While the risk for adults remained below the threshold of 1 across all simulations, there was a 2.53% probability that the HI for children could exceed 1 (Figure 9). This indicates that certain locations pose a potential health threat to children, who are more vulnerable to exposure from multiple sources than adults. An effective strategy to mitigate this risk, particularly from toxic elements like arsenic (As), is to regulate children’s contact with soil [79]. Therefore, priority for soil management and risk control should be given to the northern part of the study area, which is subject to the dual influence of a high geological background (basalt distribution) and significant contributions from coal combustion sources.

## 4. Conclusions

This study systematically investigated the spatial distribution, sources, and health risks of soil PTEs in a typical area with a high geological background in the Shandong Peninsula. The main conclusions are as follows: The mean concentrations of Cr, Cu, Ni, and Pb were found to exceed their respective background values for Chinese soils. Source apportionment using the PMF model, combined with spatial and comparative analysis, identified four primary sources: natural sources (36.79%) constituted the largest contribution, followed by a mixed traffic and agricultural source (34.20%), coal combustion (17.32%), and industrial activities (11.69%). Furthermore, land use was identified as a significant factor influencing PTE accumulation. Specifically, agricultural activities, particularly in apple orchards, led to elevated Cu levels, while industrial activities were the primary cause of Hg enrichment. The spatial patterns of Cr, Cu, and Ni were primarily controlled by parent rock materials (derived from basalt), as were those of Pb and As (derived from carbonate rocks). Finally, the health risk assessment revealed that while the non-carcinogenic risks for both children and adults were within acceptable limits, there was a 2.53% probability of unacceptable risk for children. This risk was primarily attributable to the coal combustion source, due to the high toxicity of associated elements like As. Collectively, these findings provide detailed insights into the superposition effect of natural geogenic backgrounds and anthropogenic activities on PTE accumulation in soils.

## Figures and Tables

**Table 1:** Statistical characteristics of potential toxic elements (PTEs) in soil (*n* = 19,484).

Parameters		Cd	Cr	Cu	Ni	Pb	Zn	As	Hg	pH
Mean (mg/kg)		0.13	85.0	26.0	36.2	26.8	68.4	7.6	0.03	6.58
Min. (mg/kg)		0.03	5.0	3.5	3.3	3.7	7.5	0.4	0.003	4.1
Max. (mg/kg)		6.80	1641.5	870.0	728.8	948.5	790.4	1880.2	4.22	8.1
SD		0.1	57.66	16.85	29.26	21.73	28.11	15.83	0.06	0.7
CV%		76%	68%	65%	81%	81%	41%	208%	192%	11%
BV ^a^ [37] (mg/kg)		0.097	61.0	22.6	26.9	26	74.2	11.2	0.065	7.7
BV ^b^ [38] (mg/kg)		0.132	62.0	22.6	27.1	23.6	63.3	8.6	0.031	7.32
SQGs [42] (mg/kg)	ERL	1.2	81	34	21	47	150	8.2	0.15	-
	ERM	9.6	370	270	52	218	410	70	0.71	-
SV [53]	pH ≤ 5.5	0.3	150	50	60	70	200	40	1.3	-
	5.5 ≤ pH ≤ 6.5	0.3	150	50	70	90	200	40	1.8	-
	6.5 ≤ pH ≤ 7.5	0.3	200	100	100	120	250	30	2.4	-
	pH > 7.5	0.6	250	100	190	170	300	25	3.4	-

Notes: SD, standard deviation; CV, coefficient of variance; BV ^a^, background values of soil elements in China; BV ^b^, background values of soil elements in Shandong Province; SQGs, Sediment Quality Guidelines; ERL, Effects Range-Low, threshold below which adverse effects on biota are rarely observed; ERM, Effects Range-Median, threshold above which adverse effects on biota are frequently observed. SV, standard value. References for thresholds and guidelines: [37] BV ^a^; [38] BV ^b^; [42] SQGs; [53] SV.

**Table 2:** Non-carcinogenic risk of potential toxic elements from different sources to children.

Sources	Non-Cancer Hazard Quotient (HQ)								Hazard Index (HI)
	Cd	Cr	Cu	Ni	Pb	Zn	As	Hg	
Industrial	4.2 × 10^−4^	6.6 × 10^−3^	1.9 × 10^−3^	4.9 × 10^−3^	2.3 × 10^−2^	6.6 × 10^−4^	7.5 × 10^−2^	4.3 × 10^−4^	1.13 × 10^−1^
Coal-fired	4.7 × 10^−4^	7.3 × 10^−3^	2.1 × 10^−3^	5.5 × 10^−3^	2.5 × 10^−2^	7.3 × 10^−4^	9.2 × 10^−2^	3.0 × 10^−4^	1.33 × 10^−1^
Natural source	4.4 × 10^−4^	8.9 × 10^−3^	2.3 × 10^−3^	7.1 × 10^−3^	2.5 × 10^−2^	7.7 × 10^−4^	7.6 × 10^−2^	2.9 × 10^−4^	1.21 × 10^−1^
Traffic-agricultural source	4.8 × 10^−4^	6.6 × 10^−3^	2.2 × 10^−3^	5.8 × 10^−3^	2.7 × 10^−2^	7.8 × 10^−4^	8.2 × 10^−2^	3.1 × 10^−4^	1.25 × 10^−1^
Total	1.8 × 10^−3^	3.0 × 10^−2^	8.4 × 10^−3^	2.3 × 10^−2^	9.9 × 10^−2^	2.9 × 10^−3^	3.3 × 10^−1^	1.3 × 10^−3^	4.96 × 10^−1^

**Table 3:** Non-carcinogenic risk of potential toxic elements from different sources to adults.

Sources	Non-Cancer Hazard Quotient (HQ)								Hazard Index (HI)
	Cd	Cr	Cu	Ni	Pb	Zn	As	Hg	
Industrial	1.6 × 10^−4^	1.3 × 10^−2^	2.2 × 10^−4^	5.8 × 10^−4^	2.9 × 10^−3^	8.0 × 10^−5^	8.6 × 10^−3^	6.4 × 10^−5^	2.56 × 10^−2^
Coal-fired	1.7 × 10^−4^	1.4 × 10^−2^	2.4 × 10^−4^	6.5 × 10^−4^	3.2 × 10^−3^	8.9 × 10^−5^	1.1 × 10^−2^	4.5 × 10^−5^	2.94 × 10^−2^
Natural	1.8 × 10^−4^	1.7 × 10^−2^	2.7 × 10^−4^	8.4 × 10^−4^	3.1 × 10^−3^	9.5 × 10^−5^	8.8 × 10^−3^	4.3 × 10^−5^	3.03 × 10^−2^
Traffic-agricultural source	1.7 × 10^−4^	1.3 × 10^−2^	2.5 × 10^−4^	6.8 × 10^−4^	3.3 × 10^−3^	9.4 × 10^−5^	9.3 × 10^−3^	5.1 × 10^−5^	2.68 × 10^−2^
Total	6.8 × 10^−4^	5.7 × 10^−2^	9.8 × 10^−4^	2.8 × 10^−3^	1.3 × 10^−2^	3.6 × 10^−4^	3.7 × 10^−2^	2.0 × 10^−4^	1.12 × 10^−1^

## Data Availability

The original contributions presented in this study are included in the article. Further inquiries can be directed to the corresponding authors.

## Supplementary Materials

The following supporting information can be downloaded at: https://www.mdpi.com/article/10.3390/toxics13110945/s1, Table S1: Input values of parameters for exposure dose assessment; Table S2: Values of reference dose for heavy metals; Table S3: Probability density functions (PDFs) of parameters and point value in health risk assessment with Monte Carlo simulator; Table S4: Probability density functions (PDFs) heavy metals concentration from each source. References [79,80,81,82,83,84,85,86,87,88,89,90,91,92,93,94] are cited in the supplementary materials.

## Abbreviations

The following abbreviations are used in this manuscript:

PTEs	Potential toxic elements
PMF	Positive matrix factorization
CF	Contamination Factor
SQGs	Sediment quality guidelines
ERM	Effects Range-Median
ERL	Effects Range-Low
ANOVA	Analysis of variance
