# Supplementary material for: Spatial Analysis, Influencing Factors, and Source-Oriented Probabilistic Health Risks of Potential Toxic Elements in High Geological Background Soil in Central and Southern Shandong Peninsula, China
Source: Toxics. 2025 Nov 3;13(11):945. doi: 10.3390/toxics13110945 (PMC12656397; doi:10.3390/toxics13110945)
Supplement: Supplementary file 1 [file toxics-13-00945-s001.zip › toxics-3947909-supplementary.pdf]

Table S1. Input values of parameters for exposure dose assessment.

Parameter	Description	Unit	Value		References
			Children	Adults	
IR_{ing}	Ingestion rate of soil	mg day^{-1}	200	100	[1]
IR_{inh}	Inhalation rate of soil	m^3 day^{-1}	7.63	12.8	[2]
IR_{diet}	Ingestion rate of wheat	g day^{-1}	73.64	140.2	[3]
	Ingestion rate of maize	g day^{-1}	16.68	23.6	
SA	Skin area available for soil contact	cm^2	1600	4350	[4]
AF	Soil-to-skin adherence factor	kg cm^{-2} day^{-1}	0.2	0.7	[5]
ABS	Absorption factor	unitless	0.001	0.001	[6]
PEF	Particle emission factor	m^3 kg^{-1}	1.36×10^9	1.36×10^9	[1]
EF	Exposure frequency	day $year^{-1}$	350	350	[6]
ED	Exposure duration	year	6	24	[6]
BW	Body weight	kg	15	70	[7]
AT	Average time	day	365ED	365ED	[8]

Table S2. Values of reference dose for heavy metals.

Parameter	Cd	Cr	Pb	Cu	Zn	Ni	References
RfD for ingestion (mg kg^{-1} day^{-1})	1.00E-03	1.50E+00	3.50E-03	4.00E-02	3.00E-01	2.00E-02	[9,10]
RfD for dermal contact (mg kg^{-1} day^{-1})	1.00E-05	6.00E-05	5.25E-04	1.20E-02	6.00E-02	5.40E-03	
RfD for inhalation (mg kg^{-1} day^{-1})	1.00E-05	2.86E-05	3.50E-03	4.02E-02	3.00E-01	2.01E-02	[6,11]

Note:RfD, Reference dose

Table S3. Probability density functions (PDFs) of parameters and point value in health risk assessment with Monte Carlo simulator.

Parameters	Unit	Probabilistic Distribution	Children	Adults	References
Average body weight (BW)	kg	Log-Normal	(15,1.19)	(70,1.09)	[7,12]
Exposure duration (ED)	years	Uniform	(0,6)	(0,24)	[6,13,14]
Exposed skin area (SA)	cm^2	Point	1600	4350	[4]
Skin adherence factor (AF)	mg/(cm^2•d)	point	0.2	0.7	[5]
Ingestion rate (IngR)	mg/day	Log-Normal	(100, 1.08)	(201, 1.18)	[1,12]
Average time of exposure (AT)	day	point	365 × ED	365 × ED	[8,15]
Exposure frequency (EF)	day/year	Triangular	350 (180,365)	350 (300,365)	[12,15]
Dermal adsorption factor (ABF)	dimensionles s	point	0.001	0.001	[6,15]

Notes: a1 and a2 in Log-Normal (a1, a2) defines the average value and the standard deviation for logarithmic normal distribution; b1 and b2 in Uniform (b1, b2) defines the minimum and the maximum for uniform distribution; c1, c2 and c3 in Triangular c1(c2, c3) defines the most likely value, the minimum and the maximum for triangular distribution.

Table S4. Probability density functions (PDFs) heavy metals concentration from each source.

Elements	Factor I	Factor II	Factor III	Factor IV
Cd	LN (0.028,0.029)	LN (0.031,0.028)	LN (0.032,0.030)	LN (0.033,0.028)
Cr	LN (18.24,14.42)	LN (20.28,16.35)	LN (24.72,30.29)	LN (21.77,16.26)
Cu	LN (5.77,4.64)	LN (6.37,6.24)	LN (7.15,7.30)	LN (6.75,4.75)
Ni	LN (7.66,6.31)	LN (8.54， 7.20)	LN (10.97， 16.87)	LN (9.06,6.78)
Pb	LN (6.10,7.06)	LN (6.77， 6.54)	LN (6.63,5.03)	LN (7.29,7.20)
Zn	LN (15.25,11.15)	LN (16.96， 9.98)	LN (17.97,12.23)	LN (18.21,9.76)
As	LN (1.76,5.91)	LN (2.15,3.60)	LN (1.78,5.38)	LN (1.91,5.39)
Hg	LN (0.010,0.044)	LN (0.007,0.007)	LN (0.007,0.018)	LN (0.007,0.010)

Notes: n/a not available; a1 and a2 in LN (a1, a2) defines the average value and the standard deviation for logarithmic normal distribution. .

References

1. USEPA (The United States Environmental Protection Agency), **2001**. Supplemental guidance for developing soil screening levels for superfund sites. OSWER9355.4-24. Office of Solid Waste and Emergency Response. US Environmental Protection Agency. Washington, DC.
2. Qing, X.; Yutong, Z.; Shenggao, L. Assessment of heavy metal pollution and human health risk in urban soils of steel industrial city (Anshan), Liaoning, Northeast China. *Ecotoxicology and Environmental Safety* **2015**, *120*, 377-385, doi:https://doi.org/10.1016/j.ecoenv.2015.06.019.
3. MEPPRC (Ministry of Environmental Protection of the People's Republic of China) and MLRPRC (Ministry of Land and Resources of the People's Republic of China), **2014**. Bulletin on National Survey of Soil Contamination (in Chinese). Available at: http://www.zhb.gov.cn/gkml/hbb/qt/201404/t20140417_270670.htm.
4. Environmental site assessment guideline, **2009**. DB11/T656-2009. (In Chinese).
5. USEPA (United States Environmental Protection Agency), **1993.** Reference Dose (RfD): Description and Use in Health Risk Assessments. Background Document 1A. Integrated risk information system (IRIS).
6. USEPA (The United States Environmental Protection Agency), **2011**. Exposure Factors Handbook 2011 Edition (Final). U.S. Environmental Protection Agency, Washington, DC, EPA/600/R-09/052F.
7. USEPA (The United States Environmental Protection Agency), 1991. CASRN 7440-50-8US EPA, S., **1999**. Soil Screening Guidance: Technical Background Document | Superfund | US EPA.
8. USEPA (The United States Environmental Protection Agency), **1989**. Risk assessment guidance for superfund. In: Human Health Evaluation Manual (part A). EPA/540/1-89/002. vol. 1. Environmental Protection Agency, Washington, DC.
9. USEPA (United States Environmental Protection Agency), **2015**. Risk based screening table-generic, summary table. United States Environmental Protection Agency. URL http://www.epa.gov/risk/risk-based-screening-table-generic-tables Accessed 31.01. 2016.
10. Yang, S.; He, M.; Zhi, Y.; Chang, S.X.; Gu, B.; Liu, X.; Xu, J. An integrated analysis on source-exposure risk of heavy metals in agricultural soils near intense electronic waste recycling activities. *Environment International* **2019**, *133*, 105239, doi:https://doi.org/10.1016/j.envint.2019.105239.
11. Jiang, Y.; Chao, S.; Liu, J.; Yang, Y.; Chen, Y.; Zhang, A.; Cao, H. Source apportionment and health risk assessment of heavy metals in soil for a township in Jiangsu Province, China. *Chemosphere* **2017**, *168*, 1658-1668, doi:https://doi.org/10.1016/j.chemosphere.2016.11.088.
12. Guo, G.H.; Zhang, D.G.; Wang, Y.T. Probabilistic Human Health Risk Assessment of Heavy Metal Intake via Vegetable Consumption around Pb/Zn Smelters in Southwest China. *International Journal of Environmental Research and Public Health* **2019**, *16*, doi:10.3390/ijerph16183267.
13. Men, C.; Wang, Y.; Liu, R.; Wang, Q.; Miao, Y.; Jiao, L.; Shoaib, M.; Shen, Z. Temporal variations of levels and sources of health risk associated with heavy metals in road dust in Beijing from May 2016 to April 2018. *Chemosphere* **2021**, *270*, 129434, doi:https://doi.org/10.1016/j.chemosphere.2020.129434.
14. Yang, S.; Zhao, J.; Chang, S.X.; Collins, C.; Xu, J.; Liu, X. Status assessment and probabilistic health risk modeling of metals accumulation in agriculture soils across China: A synthesis. *Environment International* **2019**, *128*, 165-174, doi:https://doi.org/10.1016/j.envint.2019.04.044.
15. Huang, J.; Wu, Y.; Sun, J.; Li, X.; Geng, X.; Zhao, M.; Sun, T.; Fan, Z. Health risk assessment of heavy metal(loid)s in park soils of the largest megacity in China by using Monte Carlo simulation coupled with Positive matrix factorization model. *Journal of Hazardous Materials* **2021**, *415*, 125629, doi:https://doi.org/10.1016/j.jhazmat.2021.125629.
